# 2′-Fucosyllactose Increases the Abundance of *Blautia* in the Presence of Extracellular Fucosidase-Possessing Bacteria

**DOI:** 10.3389/fmicb.2022.913624

**Published:** 2022-06-02

**Authors:** Ayako Horigome, Nanami Hashikura, Keisuke Yoshida, Jin-zhong Xiao, Toshitaka Odamaki

**Affiliations:** Next Generation Science Institute, R&D Division, Morinaga Milk Industry Co., Ltd., Kanagawa, Japan

**Keywords:** 2′-fucosyllactose, *Blautia wexlerae*, extracellular fucosidase, intracellular fucosidase, *Bifidobacterium pseudocatenulatum*, *Bifidobacterium bifidum*

## Abstract

*Blautia* is a genus of anaerobic bacteria that is widely distributed in the mammalian gut. Recently, an increasing body of research has demonstrated a link between this genus and human health, suggesting applications as a novel probiotic strain. Moreover, we have previously shown that 2′-fucosyllactose (2′-FL), a major component of human milk oligosaccharides, increases the relative abundance of *Blautia* sp., particularly *Blautia wexlerae*, in the cultured fecal microbiota of healthy adults using a pH-controlled single-batch fermenter. However, the effects of 2′-FL on *Blautia* proliferation vary among individuals. In this study, we assessed the impact of the intrinsic gut microbiota on the prebiotic effects of 2′-FL. Metagenomic analysis of feces collected from all donors showed that the homolog of the intracellular GH95 α-l-fucosidase gene was considerably enriched in two non-responders (individuals who showed no increase in *Blautia* proliferation), whereas the homologous genes encoding extracellular α-l-fucosidase were more abundant in responders, suggesting that lactose and fucose released into the environment could be substrates mediating the growth of *Blautia*. *In vitro* assays confirmed the ability of *B. wexlerae* to utilize the two carbohydrates but not 2′-FL. We also observed that *B. wexlerae* utilized fucose released from 2′-FL by *Bifidobacterium bifidum*, which possessed extracellular GH95 α-l-fucosidase, in co-cultures of these two organisms. Finally, increasing the proportion of extracellular GH95 by the addition of a *B. bifidum* strain led to *Blautia* proliferation by 2′-FL in fecal cultures of the two non-responders. These findings provided valuable perspectives on individualized nutritional approaches to properly control the gut microbiota. Future clinical trials are needed to obtain further insights into the characteristics of responders vs. non-responders.

## Introduction

Recent studies have shed light on the impact of alterations in the gut microbiota on human health. As our understanding of the link between the gut microbiota and host health or disease status increases, there is a greater need to incorporate methods for modulating the gut microbiota to maintain health or treat diseases. The gut microbiota is affected by various factors, including age, medications, and lifestyle. Among these factors, aging is strongly associated with the gut microbiota composition (Odamaki et al., 2016; Scepanovic et al., 2019). We previously investigated changes in the gut microbiota composition with age in healthy Japanese individuals ages 0–104 years old (Odamaki et al., 2016). The results showed that aging was accompanied by increased proportions of *Bacteroides*, *Eubacterium*, and Clostridiaceae and decreased proportions of *Bifidobacterium*, which were enriched in infants, and Lachnospiraceae, including the genus *Blautia*, which was enriched in adults. Furthermore, the metabolites reported as risk factors for age-associated diseases were enriched in the feces of elderly individuals with a distinct elderly-type gut microbiota compared with those in individuals with an adult-type gut microbiota (Yoshimoto et al., 2021). The abundances of *Blautia wexlerae* and *Bifidobacterium pseudocatenulatum* were significantly higher in the adult-type gut microbiota, whereas higher abundances of facultative anaerobic bacteria, such as *Escherichia coli*, were observed in the elderly-type gut microbiota. These findings suggest that the risk of age-associated diseases could be prevented by changing the gut microbiota from the elderly-type to the adult-type through the enrichment of bacterial taxa known to decline with age.

*Blautia* is a genus of Gram-positive, obligate anaerobic bacteria belonging to the family Lachnospiraceae. This genus is widely distributed among animals (Eren et al., 2015) and is a producer of short-chain fatty acids (Liu et al., 2021). Several studies have suggested that *Blautia* sp. have pivotal roles in human health outcomes. Compared with observations in healthy individuals, the abundance of *Blautia* is significantly lower in the fecal microbiota of obese individuals (Ozato et al., 2019; Benítez-Páez et al., 2020) and in individuals with type 1 diabetes (Murri et al., 2013) or cirrhosis (Kakiyama et al., 2013). Although the causal relationship between *Blautia* abundance and disease is not yet clear, the antibacterial activity (Khattab et al., 2017; Kalyana Chakravarthy et al., 2018) and anti-inflammatory effects (Kalyana Chakravarthy et al., 2018; Benítez-Páez et al., 2020) of these microbes may contribute to human health. Accordingly, this genus may represent a novel bacterial taxon with probiotic properties (Liu et al., 2021).

As a strictly anaerobic bacterium, *Blautia* requires harsh culture conditions with rigorous operating procedures. Therefore, it is not easy to utilize bacteria belonging to this genus as commercial probiotics. Instead, prebiotic substrates for *Blautia* could have applications in health maintenance. Some food ingredients, such as fructooligosaccharides (FOS; Mao et al., 2018), lactulose (Cui et al., 2021), and Japanese koji glycosylceramide (Hamajima et al., 2016), increase the abundances of *Blautia* in mice. Furthermore, in a previous study, we found the potential prebiotic effects of 2′-fucosyllactose (2′-FL), a major component of human milk oligosaccharides (HMOs), on *Blautia*, particularly *B. wexlerae*, using fecal fermentation in a pH-controlled single-batch fermenter (Murakami et al., 2021). However, the prebiotic effects of 2′-FL on the fecal microbiota vary among individuals.

Accordingly, in this study, we investigated the mechanisms mediating the effects of 2′-FL on the composition of the gut microbiota using a metagenomic approach followed by an *in vitro* assay.

## Materials and Methods

### Participants and Procedure

This study included a secondary data analysis of a previous study in which the potential of nine substances as prebiotics was assessed using a pH-controlled single-batch fermenter as a human gut microbiota model (Murakami et al., 2021). Fecal samples were collected from seven healthy Japanese volunteers (five men and two women, designated as participants A–G) aged between 28 and 45 years old without any bowel diseases or antibiotic treatment within the last 3 months. Written informed consent was obtained from all participants. Fresh fecal samples were collected and processed as described previously (Murakami et al., 2021).

### Fecal Fermentation

Fecal samples were cultured using a pH-controlled multichannel jar fermenter (Bio Jr.8; ABLE, Tokyo, Japan) as previously described (Murakami et al., 2021). Briefly, 100 ml yeast extract, casitone, and fatty acid (YCFA) medium supplemented with 1.0% (w/v) 2′-FL (Kyowa Hakko Bio Co., Ltd., Tokyo, Japan) as the sole carbon source (YCFA_2FL) was inoculated with 100 μl of the 10 × -diluted fecal suspension with or without 1 ml *Bifidobacterium bifidum* MCC2030 culture (approximately 2 × 10^9^ CFU/ml). Each culture was collected at 0 and 24 h after fecal inoculation at 37°C. Fecal fermentations were conducted in triplicate.

### Microbiota Analysis

DNA extraction was performed using the bead-beating method as previously described (Murakami et al., 2021). PCR amplification and DNA sequencing of the V3–V4 region of the bacterial 16S rRNA gene, carried out on an Illumina MiSeq instrument (Illumina, San Diego, CA, United States), were performed as described previously (Odamaki et al., 2018). For species-level analysis, we conducted a BLAST search to identify the representive sequences of the OTUs clustered by QIIME2 against the NCBI 16S_ribosomal_RNA database. Individual species were specified only when a single bacterial species was annotated.

### Bacterial Strains and Culture Conditions

*B. wexlerae* JCM 17041^T^ and *B. wexlerae* JCM 31267 were purchased from the Japan Collection of Microorganisms (JCM; Ibaraki, Japan). *B. bifidum* MCC2030 was obtained from Morinaga Culture Collection (MCC: Kanagawa, Japan). *B. bifidum* MCC2030 was subcultured in Lactobacilli MRS broth (Becton, Dickinson and Company, Sparks, MD, United States) containing 0.05% (w/v) l-cysteine-HCl and anaerobically incubated at 37°C for 16 h using an Anaero Pack (Mitsubishi Gas Chemical, Tokyo, Japan). *B. wexlerae* strains were subcultured in YCFA medium supplemented with 0.2% each of glucose, maltose, and cellobiose (GMC) and anaerobically incubated at 37°C for 16 h using screw-top test tubes with butyl rubber inner plugs (Sanshin Industrial Co., Ltd., Kanagawa, Japan). The gas-phase portions in screw-top test tubes were replaced with CO_2_ before autoclaving (121°C, 15 min).

### Growth Assay

Screw-top test tubes containing 5 ml YCFA with GMC, 2′-FL, lactose, or fucose were autoclaved after gas replacement with CO_2_ to establish anaerobic conditions. We inoculated 1% (50 μl) of each preculture grown as described above into the tubes. After 18 h of mono- or co-culture at 37°C, precipitates and supernatants from each culture were obtained by centrifugation. All assays were performed in triplicate or in three independently repeated experiments.

### Thin-Layer Chromatography Analysis

Degraded 2′-FL and its derivatives were evaluated using TLC with precoated silica gel-60 TLC aluminum plates (Merck, Darmstadt, Germany), as previously described (Bai et al., 2017). Samples were spotted onto plates (1 μl volume), and the plates were then developed with a solvent system consisting of 2:1:1 (v:v:v) 1-butanol:acetic acid:water. The plates were then stained by spraying with diphenylamine-aniline-phosphoric acid reagent (100 ml acetone, 1 g diphenylamine, 1 ml aniline, and 10 ml phosphoric acid) and visualized by heating in a toaster oven. The reagents were purchased from Nacalai Tesque, Inc. (Kyoto, Japan), FUJIFILM Wako Pure Chemical Corp. (Osaka, Japan), or Hayashi Pure Chemical Industry, Ltd. (Osaka, Japan).

### Quantitative PCR

qPCR was performed on an ABI PRISM 7500 Fast Real-Time PCR system (Thermo Fisher Scientific, Waltham, MA, United States) with SYBR Premix Ex Taq (Takara Bio Inc., Shiga, Japan) to quantify cell numbers for *Blautia* sp., *B. bifidum*, and *Bifidobacterium catenulatum* group (*B. catenulatum* and *B. pseudocatenulatum*) and determine copy numbers of GH95 α-l-fucosidase genes. The primer sets used in this study are shown in Supplementary Table S1. Primer sets specific for the GH95 α-l-fucosidase genes MAGB_040 and MAGF_1420 were designed using Primer 3 software (v.0.4.0; Untergasser et al., 2012). PCR amplification was performed to detect the genus *Blautia* using the following program: one cycle at 95°C for 20 s; 40 cycles at 95°C for 3 s, 55°C for 20 s, and 72°C for 50 s; and a final cycle at 95°C for 15 s. For other targets, detection consisted of the following amplification program: one cycle at 95°C for 20 s, 40 cycles at 95°C for 3 s and 60°C for 30 s, and one final cycle at 95°C for 15 s. The following bacterial strains were used as standards for quantification: *Blautia producta* ATCC 27340^T^, *B. bifidum* JCM 1255^T^, and *B. pseudocatenulatum* JCM1200^T^.

### Distribution of GH95 α-l-Fucosidase Homologs in the Metagenome Data

Metagenome sequencing was performed as described previously (Murakami et al., 2021) using DNA extracted from bacterial cells in the feces of each individual. The quality-filtered reads were mapped to the characterized GH95 α-l-fucosidases listed in the CAZy database^1^ by BLASTX with cut-off values set to identity greater than 70% and coverage greater than 60%. Normalization was performed by calculating the number of mapped reads per million total reads. Extracellular fucosidases were predicted using TMHMM-2.0^2^ and SignalP-5.0.^3^

### Analysis of Metagenome-Assembled Genomes

The high-quality metagenome reads of fecal samples from participants were used for assembly with METAnnotatorX2 v2.1 (Milani et al., 2021). High-quality trimmed reads were mapped to the contigs from each assembly using Bowtie2 (version 2.3.4.1) followed by metagenome binning using MetaBAT2 v2.12.1 (Kang et al., 2019). Gene prediction of the constructed MAGs was performed using the DNA Data Bank of Japan (DDBJ) Fast Annotation and Submission Tool version 1.2.10 (Tanizawa et al., 2018). Against the predicted amino acid sequences of constructed MAGs, BLASTP was performed to search homologs of GH95 α-l-fucosidases with cut-off values set to identity greater than 50% and coverage greater than 60%. The taxonomies of MAGs encoding the homolog of GH95 α-l-fucosidases were classified using Mash (Ondov et al., 2016) with the RefSeq database.^4^ Clinker (Gilchrist and Chooi, 2021) was used for gene cluster comparison.

### Statistical Analysis

Statistical analyses were performed using EZR software ver. 1.50 (Kanda, 2013). Intergroup differences were analyzed using unpaired Student’s *t*-tests for unpaired parametric data. For all comparisons, value of *p* less than 0.05 were considered statistically significant. Intergroup differences in microbiota were analyzed using the linear discriminant analysis (LDA) effect size (LEfSe) method with default settings (Segata et al., 2011).

### Data Availability

DNA sequences corresponding to the 16S rRNA gene and metagenome data have been deposited in the DDBJ. Accession numbers are listed in Supplementary Tables S2, S3.

## Results

### Effects of 2'-FL on *Blautia* Proliferation Varied Among Individuals

**Figure 1:**
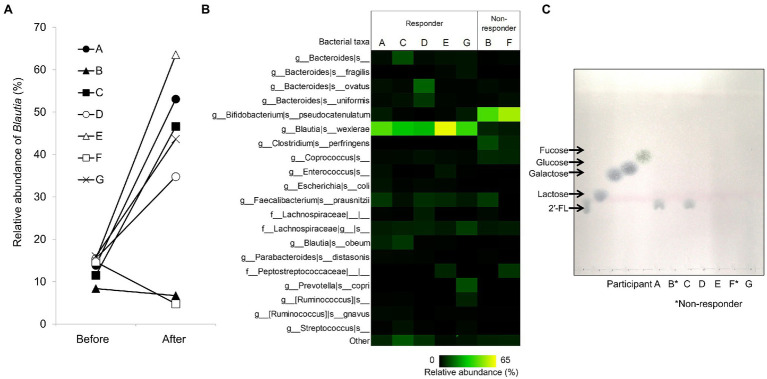
Effects of 2′-fucosyllactose (2′-FL) on fecal microbiota in a pH-controlled single-batch fermentation. **(A)** Relative abundance of *Blautia* in the fecal microbiota before and after fermentation in YCFA_2FL (*n* = 7). **(B)** Composition of the fecal microbiota for each participant after fermentation in YCFA_2FL. The relative abundances of the top 20 species-level taxa are shown; others are grouped into the “other” category. **(C)** TLC of 2′-FL utilization in fecal cultures for each participant after fecal fermentation in YCFA_2FL. Responder: participants with increased *Blautia* after fermentation in YCFA_2FL. Non-responder: participants without increased *Blautia* after fermentation in YCFA_2FL. 2′-FL, 2′-fucosyllactose and YCFA_2FL, YCFA medium supplemented with 1.0% (w/v) 2′-fucosyllactose as the sole carbon source.

We previously evaluated the effects of some prebiotic ingredients on the composition of fecal microbiota using a pH-controlled single-batch fermenter (Murakami et al., 2021). Before fermentation, *Blautia* was detected in all samples collected from seven individuals, with a median relative abundance of 14.7%. We observed a marked increase in *Blautia*, particularly *B. wexlerae*, after fecal fermentation in YCFA_2FL medium among five of the seven individuals (quartile range: 43.6–53.1), but a decrease in the other two individuals (quartile range: 5.2–6.2; Figure 1A). By contrast, the relative abundance of *B. pseudocatenulatum* was enriched in these two individuals (Figure 1B). Hereafter, we defined individuals whose fecal *Blautia* abundances were increased by 2′-FL as “responders” (participants A, C, D, E, and G), and others as “non-responders” (participants B and F). TLC analysis showed that 2′-FL was completely consumed in the two non-responders as well as in some of the responders during the fecal fermentation (Figure 1C). These data suggest that differences in *Blautia* proliferation between responders and non-responders were not related to the 2′-FL utilization ability of the fecal microbiota.

### Distribution of GH95 α-l-Fucosidase Genes in the Fecal Metagenome

**Figure 2:**
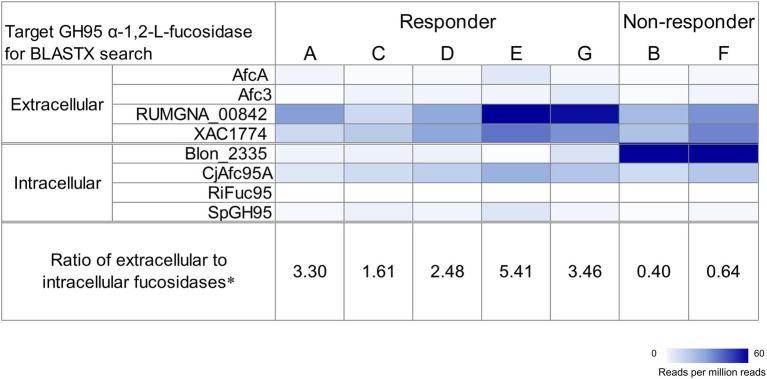
Distribution of GH95 α-l-fucosidase homologs in the fecal metagenome of each participant before fermentation. Target proteins were AfcA in *Bifidobacterium bifidum* JCM 1254, Afc3 in *Clostridium perfringens* ATCC 13124^T^, RUMGNA_00842 in *[Ruminococcus] gnavus* ATCC 29149^T^, XAC1774 in *Xanthomonas citri* pv. citri str. 306, Blon_2335 in *Bifidobacterium longum* subsp. *infantis* ATCC 15697^T^, CjAfc95A in *Cellvibrio japonicus* Ueda107, RiFuc95 in *Roseburia inulinivorans* DSM 16841^T^, and SpGH95 in *Streptococcus pneumoniae* TIGR4. The heatmap shows counts of mapped reads to target proteins per million reads. Extracellular α-l-fucosidases were predicted using TMHMM-2.0 and SignalP-5.0. *the ratio of reads mapped to the extracellular GH95 fucosidase to those mapped to the intracellular one.

Because GH95 α-l-fucosidases have been reported to play important roles in 2′-FL utilization (Katayama et al., 2004), we determined the distributions of the homologous genes of this enzyme in the fecal metagenome data before fermentation. Homologous genes for both extracellular and intracellular GH95 fucosidases were detected in all participants (Figure 2). However, homologs of the intracellular GH95 fucosidase gene Blon_2335 were considerably more abundant than those of extracellular fucosidase genes in non-responders, whereas the opposite was true for responders. In particular, homologous genes of extracellular GH95 fucosidase RUMGNA_00842 were predominant in the responders. We calculated the ratio of reads mapped to the extracellular GH95 fucosidase to those mapped to the intracellular GH95 fucosidase to assess the balance between the two. The ratio in the non-responders was less than 1, whereas that in the responders was greater than 1.61 (Figure 2).

We constructed MAGs from the fecal metagenome data before fermentation and found a MAG corresponding to *B. pseudocatenulatum*, with Blon_2335 homologous genes designated as MAGB_040 and MAGF_1420, respectively, in both non-responders (Supplementary Figure S1). The gene cluster encoding MAGB_040/MAGF_1420 contained fucosyllactose transporter genes, which were homologs of Blon_2202, Blon_2203, and Blon_2204. The copy numbers of MAGB_040/MAGF_1420 and the cell numbers of *B. pseudocatenulatum* in the culture medium were comparable after the fermentation of feces from non-responders in YCFA_2FL (Supplementary Figure S2). A MAG corresponding to *B. pseudocatenulatum* with a Blon_2335 homologous gene was not found in the fecal metagenome of the responders except for participant G. The BLASTP search of extracellular GH95 fucosidase in MAGs constructed from all individuals revealed MAGs encoding homologous genes for RUMGNA_00842 (Supplementary Table S4). Those MAGs were mainly classified in the genus *Ruminococcus*. We failed to detect any MAGs encoding extracellular fucosidase in participant C (responder).

### 2'-FL Cross-Feeding Between *Blautia wexlerae* and an Extracellular GH95 α-l-Fucosidase-Possessing Bacteria

**Figure 3:**
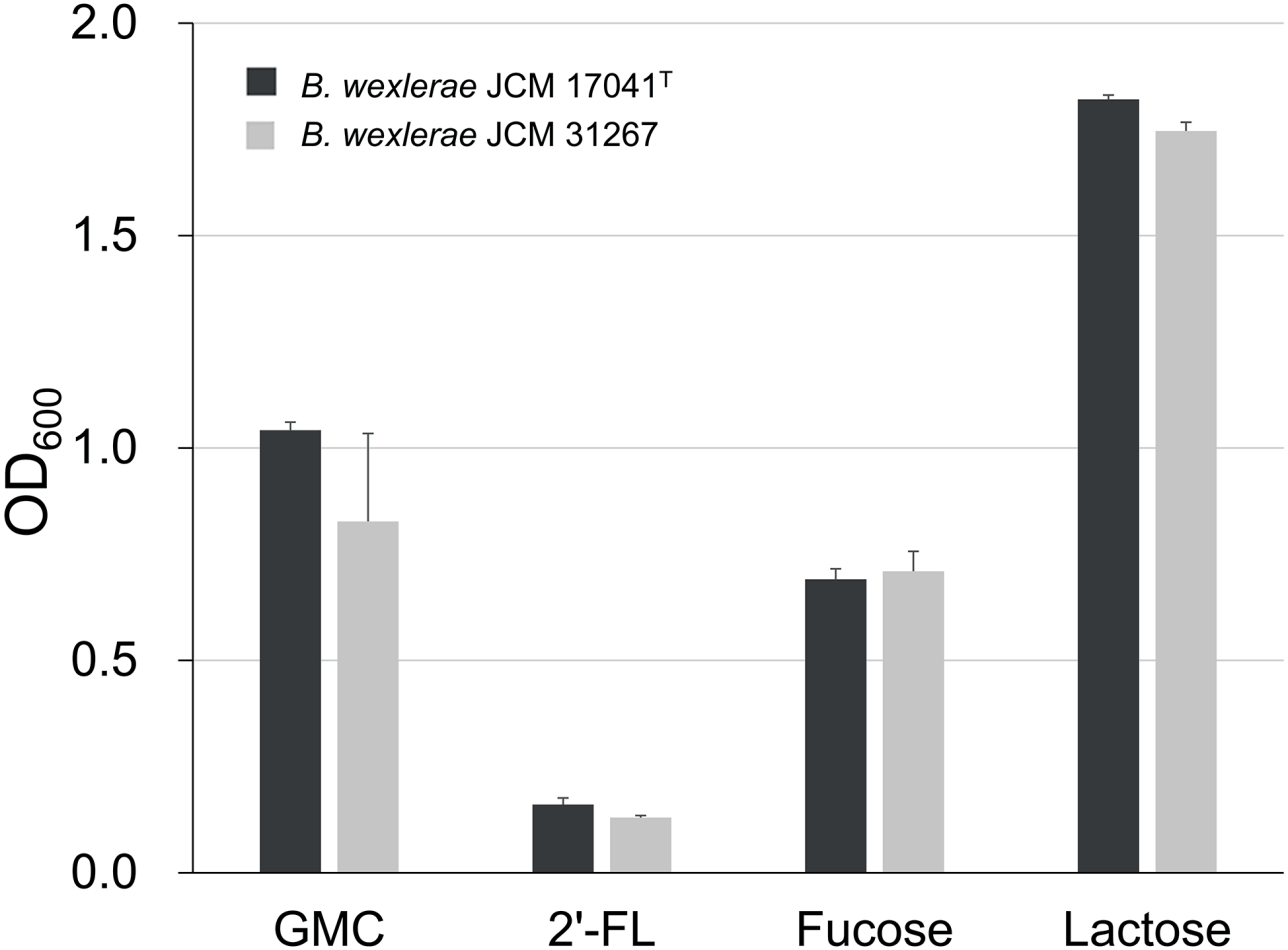
Growth of *Blautia wexlerae* in monocultures in yeast extract, casitone, and fatty acid (YCFA) for 18 h. The sole carbon source in each YCFA medium is indicated below the horizontal axis. The values represent the means ± SDs of triplicate experiments. GMC, 0.2% each of glucose, maltose, and cellobiose and 2′-FL, 2′-fucosyllactose.

**Figure 4:**
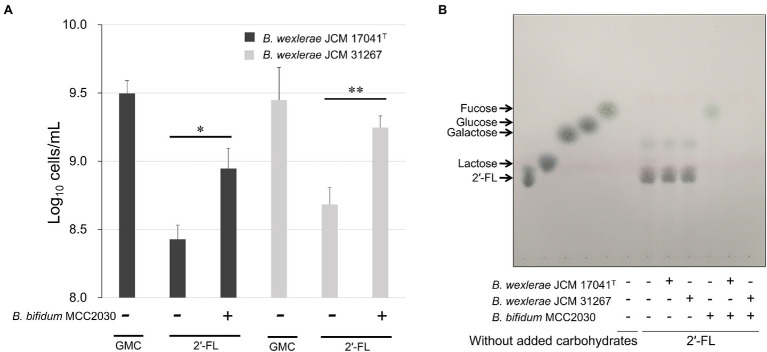
Cross-feeding of 2′-FL between *B. wexlerae* and *B. bifidum*. **(A)** Growth of *B. wexlerae* in monocultures and co-cultures with *B. bifidum* for 18 h. **(B)** TLC of 2′-FL utilization in monocultures and co-cultures of *B. wexlerae* and *B. bifidum* in YCFA_2FL. **(A)** The values represent the means ± SDs of triplicate experiments. ^*^p < 0.05, ^**^p < 0.01; detection limit: 10^6^ cells/ml. **(B)** The presence or absence of each bacterial strain and the sole carbon source in each YCFA medium are indicated below the horizontal axis. GMC, 0.2% each of glucose, maltose, and cellobiose; 2′-FL, 2′-fucosyllactose; and YCFA_2FL, YCFA medium supplemented with 1.0% (w/v) 2′-fucosyllactose as the sole carbon source.

In order to determine the 2′-FL assimilation ability in *B. wexlerae*, we performed growth assays using *B. wexlerae* strains JCM 17041^T^ and JCM 31267 in medium containing 2′-FL and its constituent sugars, fucose and lactose, respectively, as the sole carbon source. These strains showed little growth ability in YCFA_2FL, but high growth ability in YCFA with fucose and lactose (Figure 3). Subsequently, we evaluated the effects of *B. bifidum*, which has extracellular GH95 α-l-fucosidases and degrades 2′-FL extracellularly, on the ability of *B. wexlerae* strains to grow in YCFA_2FL. We used *B. bifidum* MCC2030, which had high HMO assimilation ability (data not shown). As shown in Figure 4A, the addition of *B. bifidum* MCC2030 significantly increased the growth of *B. wexlerae* strains on YCFA_2FL. Notably, fucose that was not consumed by *B. bifidum* MCC2030 in monocultures was consumed in the co-cultures (Figure 4B).

### Increasing the Proportion of Extracellular GH95 Fucosidase in Feces Led to *Blautia* Proliferation by 2'-FL in Non-responders

**Figure 5:**
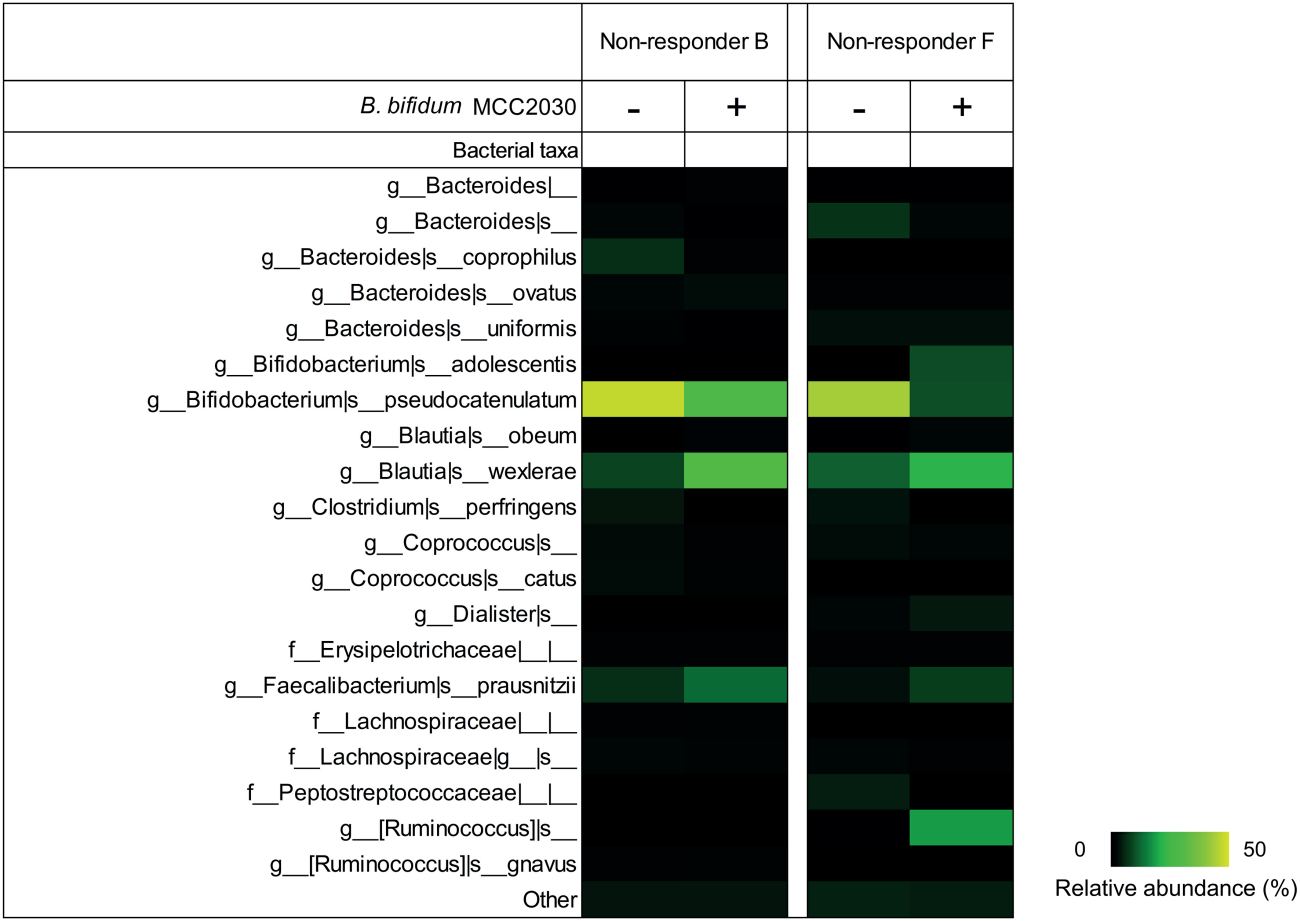
Fecal microbiota compositions of non-responders after 24 h of pH-controlled single-batch fermentation in YCFA_2FL with or without the addition of *B. bifidum* MCC2030. The relative abundances of the top 20 species-level taxa are shown; others are grouped into the “other” category. The averages of triplicate independent fermentation tests are shown. YCFA_2FL, YCFA medium supplemented with 1.0% (w/v) 2′-fucosyllactose as the sole carbon source.

**Figure 6:**
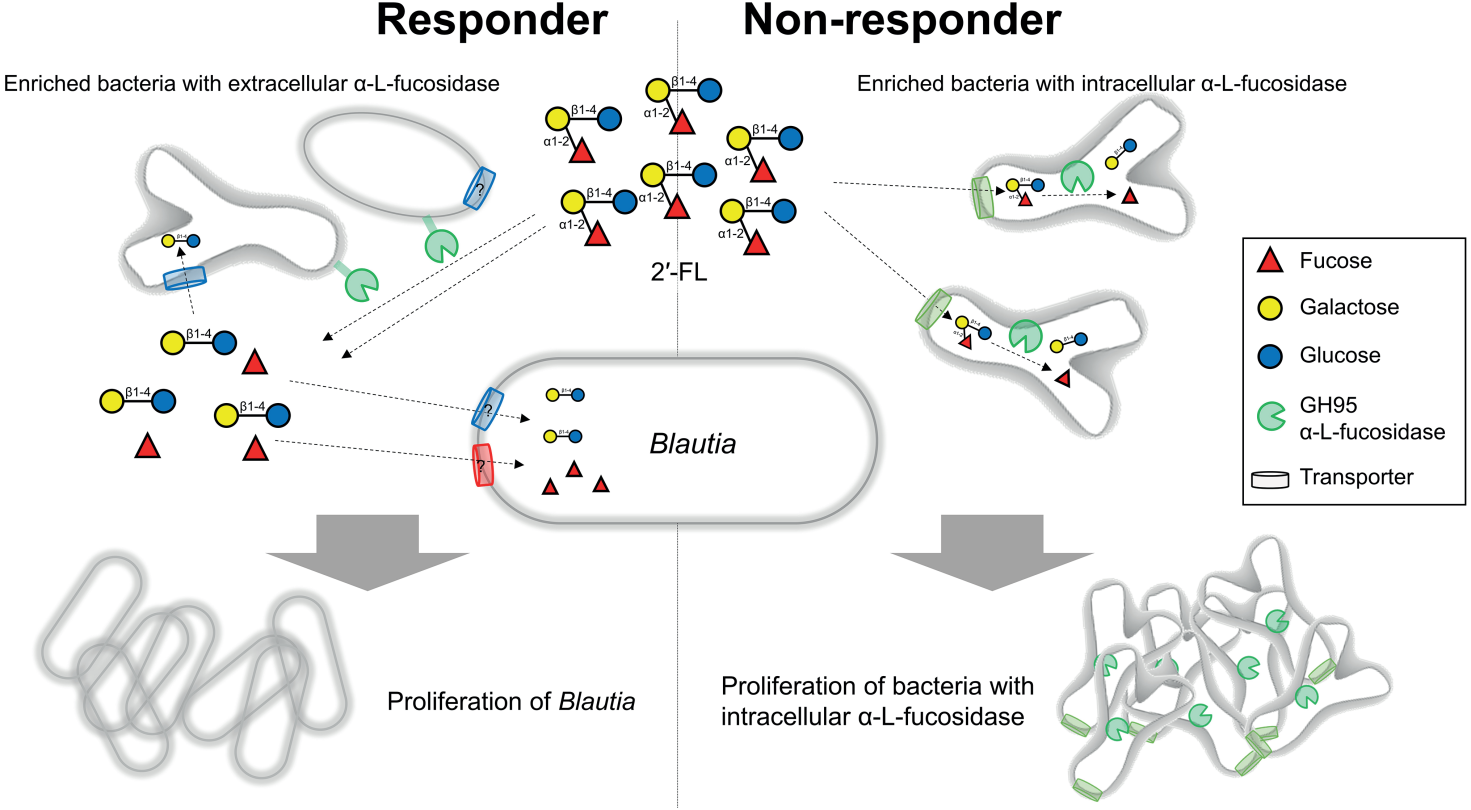
Impact of bacteria possessing extracellular or intracellular GH95 fucosidases on the prebiotic effects of 2′-FL. In responders, the increased abundance of bacteria possessing extracellular fucosidases could provide an environment rich in fucose and lactose available to *Blautia*. By contrast, an increased abundance of bacteria possessing intracellular fucosidases which directly take up and use considerable amounts of 2′-FL could result in an insufficient supply of fucose and lactose to *Blautia* in non-responders. 2′-FL, 2′-fucosyllactose.

We then assessed the effects of increasing the proportion of extracellular GH95 fucosidase on the fecal fermentation of non-responders in YCFA_2FL by the addition of *B. bifidum* MCC2030. To ensure an increase in the ratio of extracellular to intracellular fucosidase, we inoculated *B. bifidum* MCC2030 equivalent to the total number of fecal-derived bacteria into YCFA_2FL. Figure 5 shows the fecal microbiota composition of non-responders B and F after 24 h of fermentation with or without the addition of *B. bifidum* MCC2030. LEfSe analysis revealed that the addition of *B. bifidum* MCC2030 significantly decreased the relative abundances of *B. pseudocatenulatum* and increased that of *Blautia* in both non-responders B and F (Supplementary Figures S3, S4, respectively). Additionally, the addition of *B. bifidum* MCC2030 significantly increased the relative abundances of *Faecalibacterium prausnitzii* and *Collinsella aerofaciens* in both non-responders. Figure 6 shows the prebiotic effects of 2'-FL, as suggested by the findings of this study.

## Discussion

Prebiotics are promising agents with the potential to control the gut microbiota. However, previous studies have reported individual differences in the efficacy of these agents. For example, FOS or galactooligosaccharide (16 g/day) intervention in adults for 14 days was found to significantly increase the relative abundance of *Bifidobacterium*; however, no changes were observed in some individuals (Liu et al., 2017). Another prebiotics, lactulose, has also been reported to significantly increase the number of *Bifidobacterium* cells in healthy Japanese women after supplementation at 2 g/day for 2 weeks (Sakai et al., 2019). However, the response to lactulose varied among individuals, with fold changes in *Bifidobacterium* cell numbers ranging from 0.38 to 48.11. Additionally, Yoshida et al. found that the response to lactulose could be predicted by analyzing the gene encoding a solute-binding protein, which contributes to lactulose import in *Bifidobacterium*, in the baseline gut microbiota of participants (Yoshida et al., 2021). A recent review also described the importance of the genotypic composition of the resident gut microbiota. The prevalence of genes responsible for the assimilation of prebiotics would be helpful for predicting responsiveness to prebiotics (Ojima et al., 2022b). Elucidating the mechanism of individual variations in responsiveness to prebiotics may lead to the development of more effective interventions for gut microbiota control.

Our results revealed that the intrinsic gut microbiota of responders was characterized by higher amounts of extracellular GH95 α-l-fucosidases than intracellular GH95 α-l-fucosidases. Moreover, although *B. wexlerae* had limited ability to directly assimilate 2′-FL, this organism could use fucose and lactose, which are components of 2′-FL. In responders, the greater abundance of bacteria with extracellular GH95 fucosidases could provide an environment rich in fucose and lactose available to *Blautia*. On the other hand, there was an excess of intracellular GH95 fucosidase-possessing bacteria such as *B. pseudocatenulatum* in non-responders. In such cases, 2′-FL could have been directly taken up by these bacteria, which may have resulted in an insufficient supply of fucose and lactose to *Blautia* (Figure 6). Intracellular GH95 fucosidase genes are prevalent in *B. pseudocatenulatum* and play important roles in 2′-FL assimilation in this species (Shani et al., 2022; Ojima et al., 2022a). Our previous report showed that the relative abundances of *B. pseudocatenulatum* in feces were higher in non-responders B and F (24.7% and 12.2%, respectively) than in responders (0%–5.0%; Murakami et al., 2021). Fecal fermentation with an increase in the amount of extracellular GH95 fucosidases by the addition of a *B. bifidum* strain significantly increased the relative abundances of *Blautia* in non-responders. In this fecal fermentation test, the number of *B. pseudocatenulatum* in the medium before fermentation was less than 10^6^ cells/ml (Supplementary Figure S2), while that of *B. bifidum* was approximately 10^7^ cells/ml, leading to a ratio of extracellular to intracellular GH95 fucosidases of more than 10. These results suggest that the balance between extracellular and intracellular GH95 α-l-fucosidases in feces may have profound impacts on the prebiotic effects of 2′-FL.

The presence of other gut bacteria with the ability to use 2′-FL would also have an impact on the changes in the composition of gut microbiota owing to 2′-FL supplementation. In addition to *B. pseudocatenulatum*, it has been reported that *Bifidobacterium longum* subsp. *infantis* and *Bifidobacterium kashiwanohense* have the ability to take up 2′-FL directly and utilize it by intracellular degradation (Sakanaka et al., 2020). Furthermore, it has been observed that during its growth, *Bifidobacterium breve* uses fucose and lactose released by *B. bifidum* (Ward et al., 2007; Centanni et al., 2019). It has been reported that *B. pseudocatenulatum* and *B. breve* are more abundant in infants fed by secretor mothers, who secrete more fucosylated HMOs, such as 2′-FL, than in those fed by non-secretor mothers (Bai et al., 2018). Additionally, clinical studies involving infants have shown that the consumption of 2′-FL increases the abundance of *Bifidobacterium*, but not that of *Blautia* (Vandenplas et al., 2018; Berger et al., 2020). This observation could be explaind by the higher abundance of *Bifidobacerium* than that of *Blautia* in the infnat gut, similar to the non-responders in this study. It has also been demonstrated that *Bacteroides* species, including *Bacteroides thetaiotaomicron*, *Bacteroides fragilis*, and *Bacteroides vulgatus*, grow well in the presence of 2′-FL (Salli et al., 2021). However, in this study, we could not identify any impact of other 2′-FL-utilizing gut bacteria as described above, possibly because their relative abundances were much lower than those of *B. wexlerae* and *B. peudocatenulatum* or undetectable.

Recently, some commercially available synthesized HMOs, including 2′-FL, have been utilized in infant formula owing to their promising effects including prebiotic effects on *Bifidobacterium* sp. Several clinical trials have evaluated the safety, tolerance, and benefits of 2′-FL (and LNnT) in infants (Reverri et al., 2018; Vandenplas et al., 2018; Berger et al., 2020) and adults (Elison et al., 2016; Iribarren et al., 2020; Palsson et al., 2020), demonstrating modulation of the gut microbiota. Interestingly, 2′-FL and LNnT supplementation to infant formula increased the abundances of *Bifidobacterium* sp. and decreased potentially pathogenic member taxa (Vandenplas et al., 2018; Berger et al., 2020) in the gut of healthy infants. Although clinical trials evaluating the potential of HMOs in healthy adults are limited, Elison et al. reported a clinical study in which 2′-FL was administered alone to healthy adults (Elison et al., 2016). Two weeks of 2′-FL administration (5 or 10 g/day) increased Actinobacteria, to which *Bifidobacterium* belongs, and decreased Proteobacteria. The authors noted individual variations in responsiveness to 2′-FL, indicating that the initial abundance of *Bifidobacterium* determined whether an individual responded to the bifidogenic effects of 2′-FL. The abundance of *Blautia* was not changed by supplementation with 2′-FL (Elison et al., 2016). Geographic differences among individuals may have affected whether the abundance of *Blautia* was increased by 2′-FL because the human gut microbiota is diverse among populations (Nishijima et al., 2016). The gut microbiota of Japanese individuals is characterized by higher abundances of *Bifidobacterium* and *Blautia* compared with that in individuals from other countries. Therefore, clinical trials in various populations are needed to address the differences between results obtained in this study and those obtained in the previous study.

In conclusion, our results showed that the balance between extracellular and intracellular GH95 α-l-fucosidases in feces was a key factor affecting the growth of *Blautia* in medium with 2′-FL in fecal fermentation. Furthermore, it may be possible to increase *Blautia* by 2′-FL in non-responders by increasing the amount of extracellular GH95 fucosidase-possessing bacteria in the gut. Despite the strengths of this study, the findings are limited in that the sample size was very small and that we did not conduct a clinical study. Therefore, future clinical trials in which 2′-FL is administered to adults from various backgrounds will be necessary to confirm our hypothesis and elucidate the relationship between *Blautia* abundance and human health.

## Data Availability Statement

The datasets presented in this study can be found in online repositories. The names of the repository/repositories and accession number(s) can be found in the article/Supplementary Material.

## Ethics Statement

The studies involving human participants were reviewed and approved by the Ethics Committee of Japan Conference of Clinical Research. The patients/participants provided their written informed consent to participate in this study.

## Author Contributions

AH and TO conceived and designed the study and wrote the manuscript. NH conducted fecal fermentation using a pH-controlled single-batch fermenter. AH and NH performed bacterial growth assays. KY carried out the metagenomic analysis. AH, KY, J-zX, and TO interpreted the data. All authors contributed to the article and approved the submitted version.

## Conflict of Interest

AH, NH, KY, J-zX, and TO were employed by Morinaga Milk Industry Co., Ltd.

## Publisher’s Note

All claims expressed in this article are solely those of the authors and do not necessarily represent those of their affiliated organizations, or those of the publisher, the editors and the reviewers. Any product that may be evaluated in this article, or claim that may be made by its manufacturer, is not guaranteed or endorsed by the publisher.

## Footnotes

^1^ http://www.cazy.org/

^2^ https://services.healthtech.dtu.dk/service.php?TMHMM-2.0

^3^ https://services.healthtech.dtu.dk/service.php?SignalP-5.0

^4^ https://gembox.cbcb.umd.edu/mash/refseq.genomes.k21s1000.msh

## Abbreviations

2′-FL: 2′-Fucosyllactose
HMOs: Human milk oligosaccharides
YCFA_2FL: YCFA medium supplemented with 1.0% (w/v) 2′-fucosyllactose as the sole carbon source
MAGs: Metagenome-assembled genomes
LDA: Linear discriminant analysis
LEfSe: Linear discriminant analysis effect size
